# Oxidative Stress Boosts the Uptake of Cerium Oxide Nanoparticles by Changing Brain Endothelium Microvilli Pattern

**DOI:** 10.3390/antiox10020266

**Published:** 2021-02-09

**Authors:** Roberta Dal Magro, Agostina Vitali, Stefano Fagioli, Alberto Casu, Andrea Falqui, Beatrice Formicola, Lorenzo Taiarol, Valeria Cassina, Claudia Adriana Marrano, Francesco Mantegazza, Umberto Anselmi-Tamburini, Patrizia Sommi, Francesca Re

**Affiliations:** 1 BioNanoMedicine Center NANOMIB, School of Medicine and Surgery, University of Milano-Bicocca, 20900 Monza, Italy; stefano.fagioli@unimib.it (S.F.); beatrice.formicola@unimib.it (B.F.); l.taiarol@campus.unimib.it (L.T.); valeria.cassina@unimib.it (V.C.); claudia.marrano@unimib.it (C.A.M.); francesco.mantegazza@unimib.it (F.M.); francesca.re1@unimib.it (F.R.); 2 Department of Chemistry, University of Pavia, 27100 Pavia, Italy; agostina.vitali@unipv.it (A.V.); tau@unipv.it (U.A.-T.); 3 NABLA Lab, Biological and Environmental Sciences and Engineering Division, King Abdullah University of Science and Technology (KAUST), Thuwal 23955-6900, Saudi Arabia; alberto.casu@kaust.edu.sa (A.C.); andrea.falqui@kaust.edu.sa (A.F.); 4 Human Physiology Unit, Department of Molecular Medicine, University of Pavia, 27100 Pavia, Italy; patrizia.sommi@unipv.it

**Keywords:** microvilli, blood-brain barrier, cerium oxide nanoparticles, amyloid-beta, endothelial cells

## Abstract

Vascular oxidative stress is considered a worsening factor in the progression of Alzheimer’s disease (AD). Increased reactive oxygen species (ROS) levels promote the accumulation of amyloid-β peptide (Aβ), one of the main hallmarks of AD. In turn, Aβ is a potent inducer of oxidative stress. In early stages of AD, the concomitant action of oxidative stress and Aβ on brain capillary endothelial cells was observed to compromise the blood–brain barrier functionality. In this context, antioxidant compounds might provide therapeutic benefits. To this aim, we investigated the antioxidant activity of cerium oxide nanoparticles (CNP) in human cerebral microvascular endothelial cells (hCMEC/D3) exposed to Aβ oligomers. Treatment with CNP (13.9 ± 0.7 nm in diameter) restored basal ROS levels in hCMEC/D3 cells, both after acute or prolonged exposure to Aβ. Moreover, we found that the extent of CNP uptake by hCMEC/D3 was +43% higher in the presence of Aβ. Scanning electron microscopy and western blot analysis suggested that changes in microvilli structures on the cell surface, under pro-oxidant stimuli (Aβ or H_2_O_2_), might be involved in the enhancement of CNP uptake. This finding opens the possibility to exploit the modulation of endothelial microvilli pattern to improve the uptake of anti-oxidant particles designed to counteract ROS-mediated cerebrovascular dysfunctions.

## 1. Introduction

Alzheimer’s disease (AD) is the most prevalent form of dementia, accounting for 50–75% of all 50 million people worldwide living with dementia in 2020 [1]. The etiology of AD is still unclear. The major pathological hallmarks of AD include senile plaques (mainly composed of aggregated amyloid-β peptide, Aβ), neurofibrillary tangles and synapse loss. The “amyloid hypothesis” has been the dominant model for several years, describing the imbalance between the production and clearance of Aβ as the causative event triggering Aβ accumulation and cognitive impairment [2,3]. Beside this hypothesis, increasing evidence recognizes the key role of cerebrovascular dysfunctions and blood–brain barrier (BBB) impairment in AD progression [4,5]. Aβ is a potent inducer of oxidative stress. Binding of Aβ to different surface receptors activates NADPH oxidase that in turn induces reactive oxygen species (ROS) generation in vascular endothelium [6,7,8]. Moreover, the deposition of soluble Aβ species (i.e., oligomers and small assemblies) in the wall of cerebral vessels causes a cascade of events, like the release of inflammatory mediators and the activation of the complement system, which are strongly intertwined with oxidative stress. Oxidative stress, known as an imbalance between free radicals and endogenous antioxidant defenses, is a crucial and early feature in the pathogenesis of AD, further contributing to BBB damage [9,10,11]. Research studies demonstrated that oxidative stress might directly contribute to the enhancement of the amyloidogenic metabolism, leading to Aβ generation, in brain capillary endothelial cells [12,13].

These two factors, Aβ production and ROS increase, may interact and amplify each other in a vicious cycle of toxicity, promoting endothelial inflammatory response, dysregulation of cerebral blood flow, local hypoxia/ischemia and the parenchymal and perivascular deposition of Aβ [9].

Therefore, reducing vascular oxidative stress could slow down AD progression. The use of antioxidant compounds for the management of neurodegenerative diseases is mainly limited by poor bioavailability, enzymatic degradation and scarce BBB crossing [14,15]. To overcome these issues, nanovectors for the delivery of antioxidants and inorganic nanoparticles (NPs) as free radical scavenger themselves have been proposed [14,16,17,18].

In this context, cerium oxide nanoparticles (CNP) have gained increasing relevance due to the promising results obtained as ROS scavenging agents [19]. Most interest is related to the self-regenerating mechanism of CNP antioxidant activity, which results from the continuous shift between the two oxidation states of Ce (Ce^3+^/Ce^4+^) on the surface of CNP, able to scavenge superoxide anions, H_2_O_2_ and peroxynitrite. By exploiting these properties, CNP act simultaneously like the endogenous antioxidant enzymes superoxide oxidase, catalase and oxidase [19,20]. Recently, CNP have shown promising results both in vitro and in vivo for the treatment of neurodegenerative disorders, including AD [21,22,23,24].

However, CNP efficacy against vascular oxidative stress has not been evaluated so far. Thus, the present study aimed to investigate in vitro the potentiality of CNP in controlling the Aβ-induced oxidative stress on human cerebral microvascular endothelial cells (hCMEC/D3).

The most important significance and novelty of this research resides in the identification of microvilli-like protrusions on microvascular endothelial cells that undergo structural and functional modifications after exposure to pro-oxidant agents (i.e., Aβ or H_2_O_2_), and can be exploited to facilitate CNP cellular uptake. These discoveries may contribute to an increase of knowledge about the Aβ-induced changes on BBB and they open the possibility to exploit microvilli-like structures to enhance the antioxidant nanoparticle delivery to the brain.

## 2. Materials and Methods

### 2.1. Materials

Cerium(IV) oxide and 1,1′-Dioctadecyl-3,3,3′,3′-Tetramethylindocarbocyanine Perchlorate (DiI) fluorescent dye was obtained from Sigma–Aldrich (St. Louis, MO, USA). Polyacrylic acid (PAA) was from Polysciences Inc. (Warrington, PA, USA). 1,1,3,3,3-hexafluoro-2-propanol (HFIP), Aβ1-40 and Aβ1-42 peptides were purchased from Sigma–Aldrich (Milan, Italy). EBM-2 basal medium was purchased from Lonza (Basel, Switzerland). Fetal bovine serum (FBS) was from Eurobio (Paris, France). Penicillin–streptomycin (P/S) solution 100× was purchased from Euroclone (Milan, Italy). Chemically defined lipid concentrate (CDLC), Hepes, basal fibroblast growth factor (bFGF), rat tail collagen type I, trypsin-EDTA solution, NuPAGE Bis-Tris (4–12%) precast gels and 4–20% Tris-Glycine gels were all supplied by Invitrogen (ThermoFisher Scientifics, Milan, Italy). Hydrocortisone and ascorbic acid were from Sigma–Aldrich (Milan, Italy). MTT was purchased from Sigma–Aldrich, LDH assay kit was from ThermoFisher Scientifics. Rabbit anti-ERM and rabbit anti-pERM antibodies were supplied by Cell Signaling Technology (Danvers, MA, USA). Mouse anti-β-actin antibody, AlexaFluor 633 phalloidin and HOECHST were from Invitrogen. Mouse anti-Aβ 1–16 antibody (clone 6E10) was purchased from BioLegend (San Diego, CA, USA). All other chemicals were of analytical grade and were obtained from either Sigma-Aldrich (St. Louis, MO, USA) or Merck (Darmstadt, Germany).

### 2.2. CNP Synthesis and Characterization

CNP were produced by direct precipitation from aqueous solution of cerium nitride and stabilized by PAA as described previously [20,25]. CNP were characterized by high-resolution transmission electron microscopy (HRTEM), X-ray diffraction (XRD) and dynamic light scattering (DLS). Samples for HRTEM analyses were prepared by placing a drop of suspension of CNP (concentration 6 mg/mL) on ultrathin carbon membrane mounted on a 400-mesh copper grid and on 2.2 cm^2^ glass microscope slides, respectively, and left to dry. HRTEM imaging was performed using a FEI Titan 80–300 Cube transmission electron microscope (Hillsboro, OR, USA), operating at an acceleration voltage of 300 kV, equipped with a S-Twin objective lens, a FEI XFEG Schottky electron source and a 2k × 2k US1000 Gatan CCD Camera.

The XRD diffractograms were acquired in θ–θ mode, with a step of 0.03° 2θ and acquisition time of 20 s per step using a Bruker D8 Advance diffractometer (Bruker Corp., Billerica, MA, USA) with a Cu anticathode (λ-Cu-Kα = 1.541838 Å) operated at 40 kV and 40 mA.

Hydrodynamic diameters were obtained by a Nano ZS90 DLS apparatus (Malvern Instruments, Malvern, UK). Average sizes, distribution widths, polydispersion index, and associated standard deviations (SDs) were obtained for each sample from three measurements performed on diluted solutions (1 mg/mL).

For the preparation of fluorescent CNP, DiI fluorescent dye was dissolved in dimethylsulfoxide (1.2 mg/mL) and then added under stirring to the 6 mg/mL CNP suspension (1:20 *v*/*v*). DiI, as a lipophilic cation, produces a non-covalent hydrophobic interaction with PAA. The CNP suspension was centrifuged at 17,000× *g* for 20 min to remove free DiI in solution, and the pellet recovered in deionized water. A 10 nm-shift in the maximum of the emission spectra between DiI in DMSO and CNP (Supplementary Data: Figure S1) was an indication of successful intercalation of the fluorophore within the PAA hydrophobic microdomains [26].

### 2.3. Preparation and Characterization of Aβ Oligomers

Aβ1-40 and Aβ1-42 oligomers were prepared as previously described [27,28]. Briefly, the peptides were solubilized in HFIP (1 mg/mL) and air-dried in a chemical fume hood overnight. To obtain monomer-enriched preparations, the peptides were resuspended in DMSO (5 mM) and bath sonicated for 10 min (Aβ1-42) or 30 min (Aβ1-40). Then, samples were diluted in PBS at a final concentration of 100 μM of Aβ1-42 or Aβ1-40 and incubated for 24 h at 4 °C to obtain oligomeric Aβ preparations. The morphology of Aβ oligomers was assessed by atomic force microscopy (AFM), as described [29]. The aggregation state of the samples was evaluated by SDS-PAGE gel electrophoresis on a 4–20% Tris-Glycine gel, followed by immunoblotting analysis using 6E10 anti-Aβ antibody (1:1000). Aβ assemblies were visualized with enhanced chemiluminescence system by Amersham Imager 600 (GE Healthcare Srl, Milano, Italy).

### 2.4. Cells and Culture Conditions

Immortalized human cerebral microvascular endothelial cells (hCMECs) were provided by Dr. S. Bourdoulous (Institut Cochin, Inserm, Paris, France) and used as a model of the brain capillary endothelium [30]. Cells at passage between 25 and 35 were seeded on tissue culture flasks, pretreated with rat tail collagen type I (0.05 mg/mL). Cells were grown in complete culture medium (EBM-2 supplemented with 10% FBS, 1% CDLC, 1% P/S, 10 mM Hepes, 5 µg/mL ascorbic acid, 1 ng/mL bFGF and 1.4 µM hydrocortisone) and maintained at 37 °C, 5% CO_2_. Culture medium was changed every 2 days.

### 2.5. Cell Viability in the Presence of CNP or Aβ Oligomers

hCMEC/D3 cells were cultured on collagen-coated 96-wells plates (3 × 10^4^ cells/cm^2^) for 48 h. To assess the biocompatibility of CNP, cells were incubated with different concentrations of CNP (ranging from 50 and 500 µg/mL) in complete cell culture medium, for 3 h and 24 h. At the end of treatment, cell viability was evaluated by MTT and LDH assays as previously described [31] using a microplate reader (SPECTROstar Nano, BMG LABTECH, Ortenberg, Germany). Cell viability after exposure to Aβ oligomers was also assessed. Briefly, hCMEC/D3 cells were incubated with 0.1, 1 and 10 µM of Aβ oligomers in the complete cell culture medium. After 24 h, the mitochondrial activity was measured by MTT assay.

### 2.6. Free Radical Scavenging Activity of CNP after hCMEC/D3 Exposure to Aβ Oligomers

hCMEC/D3 cells were seeded on collagen-coated 35 mm dishes at a density of 3.5 × 10^4^ cells/cm^2^ and incubated at 37 °C, 5% CO_2_ for 48 h. Then, cells were incubated with 1 µM of Aβ oligomers in complete medium without FBS in order to prevent the Aβ sequestering by serum proteins [32]. Different conditions were set up according to the following scheme: (i) incubation with Aβ for 4 h; (ii) incubation with Aβ for 24 h; (iii) incubation with Aβ for 1 h, addition of CNP (50 µg/mL) to the medium and further incubation for 3 h and (iv) incubation with Aβ for 21 h, addition of CNP (50 µg/mL) to the medium and further incubation for 3 h. At the end of the incubations, cells were washed twice with PBS, detached using a scraper and sonicated on ice. Then, the samples were centrifuged at 12,000× *g* for 10 min at 4 °C and the supernatants were collected. The presence of ROS/RNS in the supernatants was detected using the Oxiselect In Vitro ROS/RNS Assay Kit (Cell Biolab, Inc., San Diego, CA, USA), according to the manufacturer’s instructions. The fluorescence intensity of dichlorodihydrofluorescein (DCF; λex = 480 nm, λem = 530 nm), which reflects the total level of ROS/RNS within the sample, was measured with Wallac 1420 Victor2 spectrofluorometer (Perkin Elmer, Waltham, MA, USA). A standard curve was obtained using scalar concentrations of DCF.

### 2.7. Uptake of CNP by hCMEC/D3 Cells

The internalization of CNP by hCMEC/D3 cells was evaluated under basal conditions and after exposure to Aβ oligomers. Cells were seeded on rat tail collagen I-coated 96-wells Cell Carrier Ultra plates (Perkin Elmer) at a density of 3 × 10^4^ cells/cm^2^. After 2 days, cells were incubated with Aβ oligomers (1 µM) for 1 h in the complete culture medium without FBS in order to prevent the Aβ sequestering by serum proteins [32]. Then, 50 µg/mL of DiI-labeled CNP (λex = 549 nm, λem = 565 nm) were added to the medium and the cells were incubated for additional 3 h in the dark. At the end of the incubation, hCMEC/D3 cells were washed with PBS and fixed with 10% formalin. Then, cells were permeabilized with 0.2% Triton (*v*/*v*) in PBS for 15 min, followed by staining of actin cytoskeleton with Phalloidin AlexaFluor 633 (1:100 in PBS) for 1 h at room temperature (RT). After washes with PBS, nuclei were counterstained with Hoescht 33342 (1:5000 in PBS) for 15 min at RT. Cells were washed with PBS and images were acquired using the Operetta CLS High Content Analysis System (Perkin Elmer) equipped with 40× water objective and standard instrument filters. Ten different fields were imaged in each well. About 400 cells for each condition were analyzed and the fluorescence intensity of CNP was measured by the Harmony analysis software (PerkinElmer). For SEM analysis, fixed samples were coated with carbon film and analyzed with a field emission scanning electron microscope (Mira3 XMU; Tescan, Kohoutovice, Czech Republic) equipped with an EDAX EDS microprobe and backscattered electron (BSE) detector. The SEM was operated at an accelerating voltage of 15kV.

### 2.8. Binding of CNP to Aβ

To evaluate if CNP were able to bind Aβ, 1 uM of Aβ oligomers were added to 50 μg/mL of CNP in PBS, in order to prevent the Aβ sequestering by serum proteins [32]. Samples were incubated for 30 min at 37 °C and gently mixed every 10 min. After incubation, samples were centrifuged at 15,000× *g* for 15 min at RT. Supernatant (representing free Aβ) and pellet (representing Aβ-bound to CNP) were collected and the Aβ content was determined by the ELISA kit assay (IBL International, Hamburg, Germany).

### 2.9. Visualization of Microvilli-Like Protrusions by SEM

hCMEC/D3 cells were grown on collagen precoated 35 mm dishes (3.5 × 10^4^ cells/cm^2^) for 2 days, followed by incubation with 1 μM of Aβ oligomers in the complete medium for 24 h. Then, cells were washed with cacodylate 0.05 M and fixed with glutaraldehyde 2.5% (*v*/*v*) in cacodylate for 90 min. After washing with cacodylate 0.05 M, samples were dehydrated in an ethanol gradient (70%, 80%, 90% and 100% *v*/*v* in water, 15 min/each), followed by chemical drying with hexamethyldisilazane (HMDS). Briefly, cells were incubated with HMDS:ethanol (1:2) for 20 min, then with HMDS:ethanol (2:1) for 20 min and finally with 100% HMDS for 20 min. Fresh HDMS was added to the dishes and evaporated in a chemical fume hood overnight.

For SEM analysis, samples were coated with carbon film and imaged using secondary electrons (SE) and backscattered electrons (BSE) detectors. Microvilli length was measured from SEM-SE images by using ImageJ software. At least 10 cells and 300–400 protrusions were analyzed for each condition. For each image, the microvilli length was obtained by using the “segmented line” tool of the program and the resulting values were exported in Excel. For each condition, microvilli were subdivided, based on the frequency analysis, in three classes in relation to their length (short: 0–0.46; medium: 0.47–1.38; long: 1.39–2.53 μm) and expressed as a percentage.

To evaluate CNP binding to microvilli-like structures, cells seeded on 35 mm dishes were treated with 1 μM of Aβ oligomers in complete medium without FBS for 24 h. Then, CNP (50 µg/mL) were added to the medium and cells were incubated for 15 min at 37 °C. Samples were fixed, dehydrated and dried as described for SEM analysis. To visualize cell-surface bound CNP, samples were imaged using BSE detectors and an accelerating voltage between 5 and 10 kV.

### 2.10. In Vitro BBB Model

The in vitro BBB model was set up as previously described, with some modifications [27,33,34]. hCMEC/D3 cells were seeded at a concentration of 5 × 10^4^ cells/cm^2^ on the apical side of Transwell inserts (polyester membrane inserts 1.12 cm^2^, pore size 0.4 μm, Greiner Bio-One, Kremsmünster, Austria) precoated with rat tail collagen type I (40 μg/cm^2^). The apical chamber (representing the blood) was filled with 500 μL of culture medium, while the basolateral chamber (representing the brain side) was filled with 1 mL of medium. hCMEC/D3 were grown for 3 days in complete cell culture medium. After 3 days, the medium was replaced with EBM-2 supplemented with 5% FBS, 1% CDLC, 1% P/S, 10 mM Hepes, 5 µg/mL ascorbic acid, 1.4 µM hydrocortisone and 10 mM LiCl. Cells were maintained at 37 °C, 5% CO_2_ and the medium was changed every 2 days.

### 2.11. Immunoblotting for pERM/ERM Levels

Six days after hCMEC/D3 seeding on the Transwell system, 0.5 mM H_2_O_2_ or different concentrations of Aβ oligomers (0.1–10 µM) were added to the apical compartment in complete culture medium. Otherwise, to assess if the expression of ezrin–radixin–moesin (ERM) protein complex was related to cell polarization, Aβ oligomers (0.1–10 µM) were incubated in the basolateral compartment. After 24 h, cells were washed with PBS, detached from the filter using trypsin-EDTA and centrifuged at 130× *g* for 5 min. The pellet was washed in cold PBS, resuspended in cold lysis buffer (50 mM Trizma, 150 mM NaCl, 2 mM EDTA, 1 mM MgCl_2_, 100 mM NaF, 10% glycerol, 1% Triton X-100, 1% sodium deoxycholate, 1% SDS, 125 mM sucrose, 1% protease and phosphatase inhibitor cocktails (Thermo Fisher Scientific)) and gently rotated for 30 min at 4 °C. After centrifugation at 13,000× *g* for 15 min at 4 °C, the supernatant was retained and total protein content was quantified by bicinchoninic acid assay (Pierce BCA Protein Assay Kit, Thermo Fisher Scientific). Of the total proteins 30 µg was separated by SDS-PAGE using 4–12% NuPAGE Bis-Tris gel and blotted onto nitrocellulose membrane. After blocking with TBS-Tween 0.1% + 5% bovine serum albumin (BSA) for 1 h at RT, membranes were immunodecorated alternatively with the primary antibodies: rabbit anti-ERM (1:1000), rabbit anti-pERM (1:1000) and mouse anti-β-actin (1:1500) in TBS-Tween 0.1% + 5% BSA overnight at 4 °C. After washes with TBS-Tween 0.1%, nitrocellulose membranes were incubated with the appropriate HRP-conjugated secondary antibody goat anti-rabbit (1:20,000) or goat anti-mouse (1:20,000) diluted in TBS-Tween 0.1% + 5% BSA for 90 min at RT. Protein bands were detected with an enhanced chemiluminescence system using Amersham Imager 600 and analyzed using NIH ImageJ software. All the data have been normalized to β-actin.

### 2.12. Statistical Analysis

Data are expressed as the mean and standard deviation (SD). For CNP uptake quantification, data were analyzed by Student’s *t*-test. For all the other experiments, data were analyzed with one-way ANOVA. Experiments were performed at least in triplicate. The analysis was performed using GraphPad Prism software. A *p*-value < 0.05 was considered statistically significant.

## 3. Results

### 3.1. Characterization of CNP

**Figure 1:**
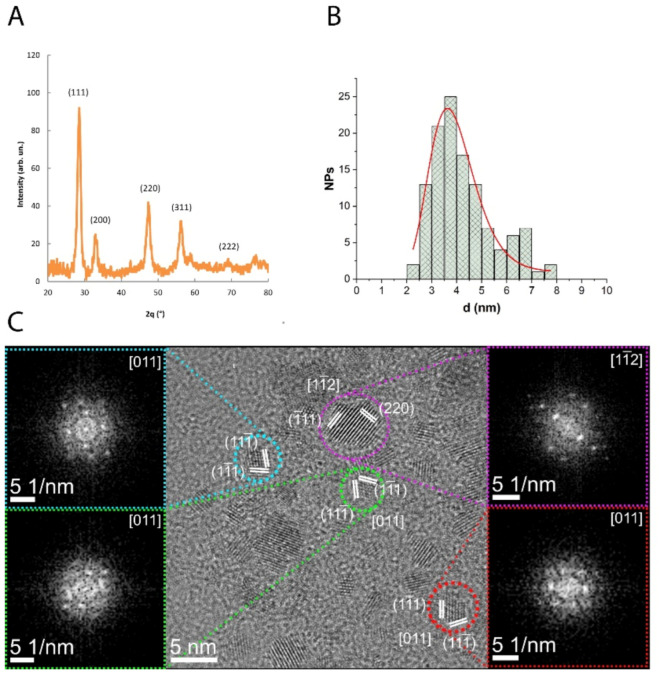
Physicochemical characterization of the synthetized CNP. (**A**) XRD pattern. (**B**) CNP diameter distribution as measured from several HRTEM images, fitted with a log-normal function (red line). (**C**) HRTEM images of representative CNP. Magnified regions show the single-crystalline structure of CNP, confirmed by the 2D-FFT diffractograms. For improved readability, only the main lattice planes are indicated in the insets.

The CNP used in this study were obtained by direct precipitation from aqueous solution and wrapped with a coating of PAA as the protective substance [20]. XRD analysis showed that all diffraction peaks corresponded to the fluoritic CeO_2_ crystal structure (PDF 98-002-8709) (Figure 1A). HRTEM and the filtered bidimensional fast Fourier transform (2D-FFT), evidenced that CNP were monocrystalline, with a crystal structure in agreement with the XRD results. Moreover, HRTEM images showed neither evidence of internal defects nor of significant agglomeration and allowed estimating the particle mean size to be about 4 nm, not accounting for the polymeric shell (Figure 1B,C). The hydrodynamic diameter determined by DLS was 13.9 ± 0.7 nm, including the polymeric shell surrounding the CNP. No significant changes in diameter were detected after CNP dilution in culture medium. These results are in accordance with previous data, where CNP were prepared following the same procedure [20].

### 3.2. Cell Viability in the Presence of CNP or Aβ Oligomers

Before testing the antioxidant properties of CNP, the experimental conditions were set up in order to preserve cell viability.

**Figure 2:**
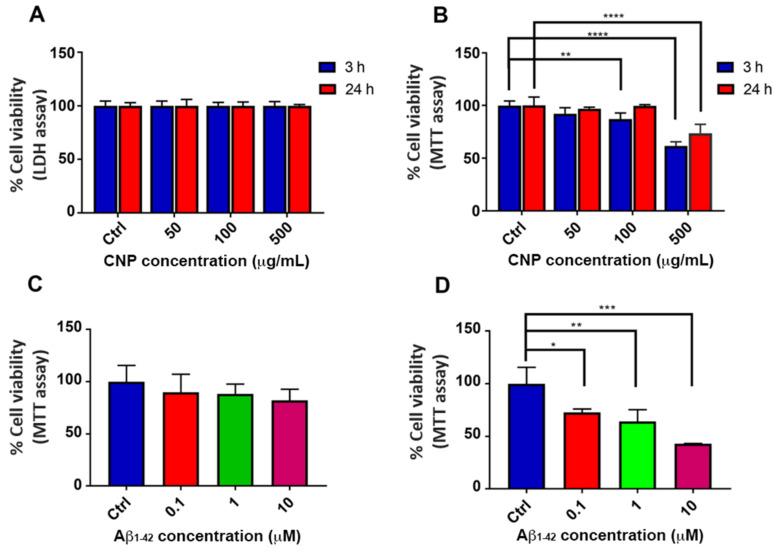
Cell viability after exposure to cerium oxide nanoparticles (CNP) and Aβ. (**A**,**B**) Human cerebral microvascular endothelial cells (hCMEC/D3) were incubated with different doses of CNP (from 50 to 500 μg/mL) for 3 and 24 h. The cytotoxicity of CNP was assessed by LDH (**A**) and MTT (**B**) assays. (**C**,**D**) hCMEC/D3 were seeded on collagen-coated 96 well plates and after 48 h of culture, cells were incubated with increasing concentrations of Aβ oligomers, ranging from 0.1 to 10 μM. After 24 h of exposure to Aβ1-42 (**C**) or Aβ1-40 (**D**), cell viability was evaluated by MTT assay. Data are expressed as percentages relative to controls (untreated cells). Bars represent the mean of 3 replicates SD. Statistical analysis was performed using one-way ANOVA: * *p* < 0.05; ** *p* < 0.01; *** *p* < 0.0001; **** *p* < 0.0001.

The viability of hCMEC/D3 cells was monitored after exposure to different doses of CNP, ranging from 50 to 500 μg/mL, using two techniques that are described in Section 2 Materials and Methods. Cell treatment with CNP did not affect cell membrane integrity at any tested concentration, as assessed by measuring LDH release (Figure 2A). On the contrary, a reduction of cell viability (assessed by MTT assay) of 13% and 38% was observed after 3 h incubation with 100 μg/mL and 500 μg/mL of CNP, respectively (Figure 2B). These findings are consistent with previous studies showing that the viability of different cell lines is preserved after treatment with CNP up to 200 μg/mL [20,35,36], while doses higher than 400 μg/mL appear to be cytotoxic by interfering with mitochondrial functions [36]. Based on these results, the lowest CNP dose tested (50 μg/mL) corresponding to 1 × 10^4^ CNP was selected for all the subsequent experiments. 

Then, we evaluated the effect of Aβ oligomers, the most toxic Aβ species [37,38,39], on hCMEC/D3 cell viability. In particular, we tested the effect of both Aβ1-42, the most abundant fragment in both amyloid brain and vascular deposits, and Aβ1-40, which is enriched in the wall of brain vessels and capillaries [40]. Aβ1-40 and Aβ1-42 oligomers were characterized by SDS-PAGE/western blot (WB) and AFM. The results showed that the preparations were enriched in oligomers (MW between 6.5 and 17 kDa), and characterized by the absence of high molecular weights aggregates (Supplementary Data: Figure S2A, lane 1 and 2). Monomer-enriched preparations were also analyzed as control (Supplementary Data: Figure S2A, lane 3 and 4). These results are consistent with the AFM images, where only small assemblies are visualized (Supplementary Data: Figure S2B,C).

The cytotoxicity of Aβ oligomers was checked by the MTT assay on cultured brain vascular endothelial cells and the results showed that after 24 h of incubation with Aβ1-42 oligomers, the cell viability was not affected even at the highest dose (Figure 2C). Conversely, hCMEC/D3 exposure to Aβ1-40 oligomers caused a dose-dependent cytotoxicity. In particular, treatment with 1 µM and 10 µM of Aβ1-40 reduced cell viability of 36% and 57%, respectively (Figure 2D). Accordingly, Xu and colleagues demonstrated that incubation of bovine and murine cerebral endothelial cells with 1–10 µM of Aβ1-40 lead to a substantial increase in cell death [39]. The differences in cell viability between Aβ1-40 and Aβ1-42 treatment could be due to the higher tendency of Aβ1-42 than Aβ1-40 to form fibrils in culture medium at 37 °C, which are known to be less toxic compared to oligomers [41].

Based on these results, 1 µM of Aβ oligomers was selected for all the subsequent experiments in order to maintain the cell viability >60%.

### 3.3. Evaluation of the Antioxidant Activity of CNP in Cerebral Endothelial Cells Exposed to Aβ

To test if cell incubation with 1 μM of Aβ oligomers induces oxidative stress, the production of ROS/RNS by hCMEC/D3 was evaluated by the DCF assay, whose fluorescence is proportional to the level of ROS and RNS species produced. After 4 h of incubation with 1 μM Aβ oligomers, a 2-fold higher ROS/RNS production was observed compared to the basal condition of untreated cells.

Prolonged exposure to Aβ up to 24 h did not further increase the DCF fluorescence, suggesting that Aβ-induced oxidative stress is time-independent. These results are comparable to previous published data showing that the treatment of HUVEC cells with Aβ25-35 induces a strong increase of H_2_O_2_ production [42]. However, at the best of our knowledge there are no data about the effect of Aβ oligomers on ROS/RNS production by cerebral microvascular endothelial cells.

**Figure 3:**
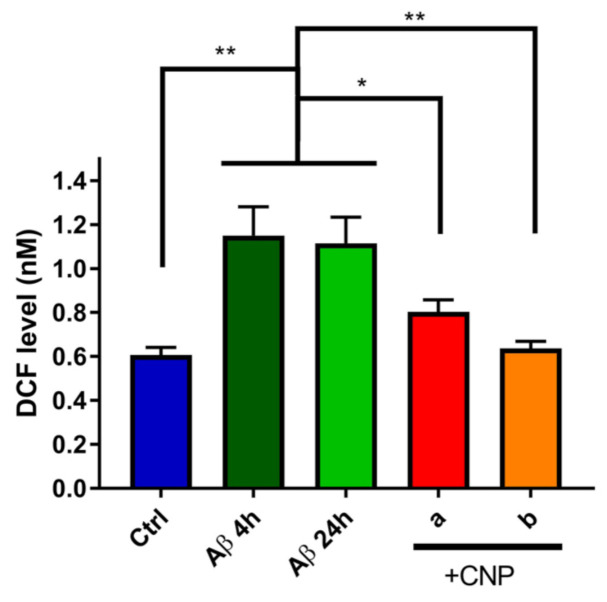
Scavenging activity of cerium oxide nanoparticles (CNP) towards Aβ-induced free radical species. The fluorescence of dichlorodihydrofluorescein (DCF), which is proportional to the level of ROS/RNS species in the samples, was measured under basal condition, after incubation with Aβ oligomers (1 μM) for 4 and 24 h and after addition of 50 μg/mL CNP (“a” = 1 h Aβ alone + 3 h in the presence of CNP; “b” = 21 h Aβ alone + 3 h in the presence of CNP). Ctrl: mean of DCF levels of untreated cells after 4 or 24 h in culture. Data are expressed as mean ± SD from three independent experiments. Statistical analysis was performed using one-way ANOVA: * *p* < 0.05; ** *p* < 0.01.

Incubation with CNP for 3 h in the presence of Aβ oligomers (as 1 h or 21 h of pretreatment, “a” and “b” respectively) leads to a significant reduction (−36% ± 6.2%) of ROS/RNS production by hCMEC/D3 cells in the tested conditions (Figure 3), thus suggesting that CNP scavenging activity is effective both as early treatment and after prolonged exposure to Aβ. This result supports the time-independent activity of Aβ in inducing ROS production.

### 3.4. CNP Cellular Uptake after Exposure to Aβ

**Figure 4:**
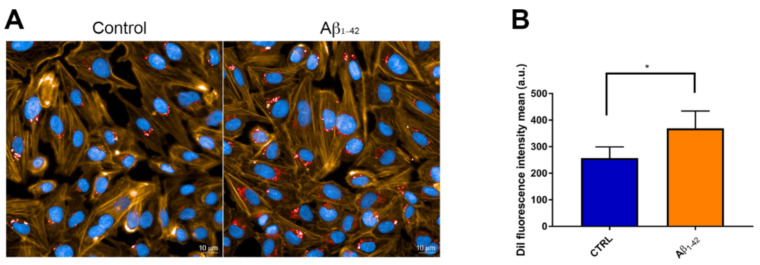
Uptake of cerium oxide nanoparticles (CNP) by hCMEC/D3 cells. (**A**) Fluorescent microscopy images show the internalization of CNP (50 μg/mL, 3 h of incubation) by hCMEC/D3 cells under basal conditions (left panel) and after 1 h of exposure to 1 μM Aβ1-42 oligomers (right panel). Red staining is DiI-labeled CNP, orange staining is phalloidin-labeled cytoskeleton and blue is nuclear staining. (**B**) Quantification of CNP-associated fluorescence in the two experimental conditions. About 400 cells were analyzed per group. Data are expressed as mean ± SD from three independent experiments. Statistical analysis was performed using a Student’s *t*-test: * *p* < 0.05.

To obtain high specificity and high resolution in the imaging of the internalized CNP, fluorescent microscopy and SEM have been combined [43]. Images (Figure 4A) showed that after 3 h of incubation, DiI-labeled CNP were internalized by hCMEC/D3 cells and gathered mainly in the perinuclear region of the cells as already reported for other NP [44,45]. Interestingly, an increased uptake of CNP (+43%) was detected in cells exposed to 1 μM Aβ oligomers, compared to untreated cells (Figure 4B).

These same observations were also confirmed by SEM analysis (Supplementary Data, Figure S3) where CNP are visible as bright spots on a darker background around cell nuclei.

Despite the negative surface charge, due to the presence of PAA, the CNP cellular internalization observed suggests that the electrostatic interactions only partly contribute to nanoparticle/cell interaction, as already shown for other negatively charged nanoparticles [44,46].

### 3.5. Pro-Oxidant Stimuli Affect the Architecture of Endothelial Microvilli

Since CNP were not surface functionalized to target BBB endothelial cells and since human endothelial cells do not possess specific receptors for CNP internalization, to gain insight into the mechanisms involved in the uptake of CNP, we explore the possibility that Aβ could act as a carrier for CNP internalization, since it is known that Aβ can be exploited to permeate the BBB [47]. Therefore, we analyzed the binding between Aβ oligomers and CNP by centrifugation and the results showed that less than 1% of total Aβ (for both fragments tested) was bound to the CNP surface, thus suggesting that CNP were uptaken in the Aβ-independent way.

**Figure 5:**
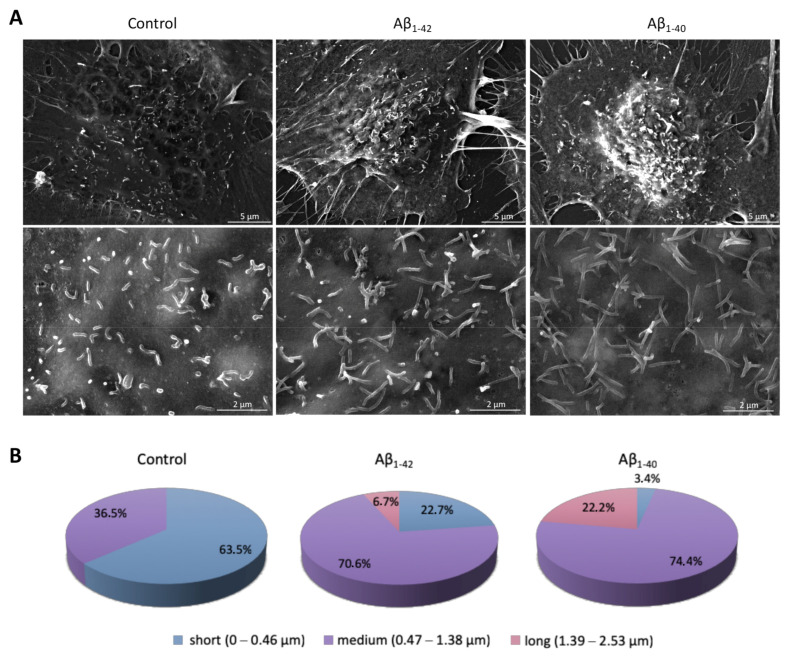
Morphological changes of endothelial microvilli after cell exposure to Aβ. (**A**) SEM-SE images of cell membrane protrusions formed by hCMEC/D3 cells under normal conditions or after incubation with 1 μM of Aβ1-42 or Aβ1-40 oligomers for 24 h. Magnified views of microvilli (30kx) are shown in the lower panels. (**B**) Classification and distribution of endothelial microvilli according to their length in the different experimental conditions. At least 10 cells and 300–400 protrusions were analyzed for each condition. Measurements were performed using ImageJ software. Control: untreated cells; Aβ1-42: cells exposed to Aβ1-42 oligomers; Aβ1-40: cells exposed to Aβ1-40 oligomers.

Then, we evaluated the possibility that Aβ could induce some structural modifications on brain endothelial cells that may enhance the internalization of CNP. SEM photograms of hCMEC/D3 cells revealed small cellular membrane protrusions covering the surface of endothelial cells (Figure 5A). These protrusions, known as endothelial microvilli-like structures, have been firstly described by Gabbiani and coworkers in rat vessels [48]. A complete elucidation about the role of these microvilli on endothelial cells is still lacking, although their involvement in blood flow dynamics [49], leukocyte recruitment [50,51] and bacterial internalization [52] has been reported. Moreover, endothelial microvilli have been described as dynamic structures whose surface density and length is affected by different conditions, including ischemia and vessel injury [53,54]. For these reasons, we assessed the architecture of these protrusions on hCMEC/D3 in our experimental conditions. Although we did not find significant changes in microvilli density on hCMEC/D3 surface after exposure to both Aβ oligomers tested (CTRL = 0.67 microvilli/μm^2^; Aβ1-42 = 0.48 microvilli/μm^2^; Aβ1-40 = 0.51 microvilli/μm^2^), interestingly, the pattern of microvilli length was different (Figure 5B). In particular, untreated cells displayed mainly short membrane protrusions with an average length of about 0.45 μm. The incubation with 1 μm Aβ1-42 oligomers for 24 h induced a strong increase of medium-length microvilli with an average length of 0.82 μm. Curiously, also the treatment with 1 μm Aβ1-40 led to a strong increase of medium-length microvilli with an average length of 1.18 μm, but with a marked reduction (>90%) of short protrusions.

The formation of microvilli requires the activation of the ERM protein complex through the phosphorylation of a threonine residue in the conserved C-terminal domain of ERM proteins [52]. The phosphorylation unmasks F-actin binding sites in the C-terminal region of the proteins, and the active form of the complex act as a linker between the cytoskeleton and the cytoplasmic tail of plasma membrane proteins (such as CD44 and ICAMs), inducing the morphogenesis of cell protrusions [55,56,57].

**Figure 6:**
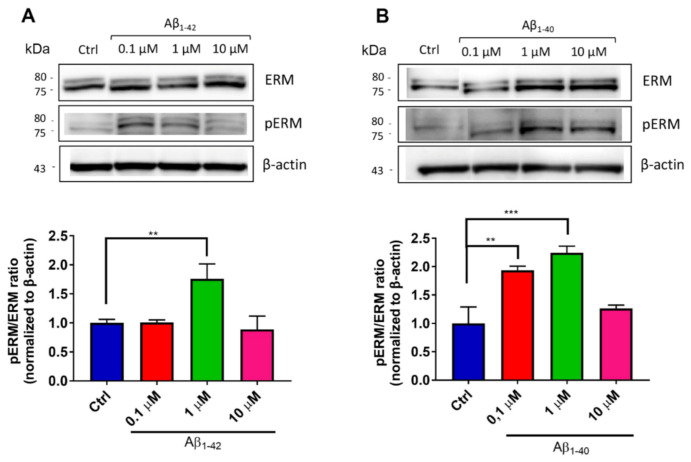
Expression of the ezrin–radixin–moesin (ERM) protein complex by hCMEC/D3 cells after exposure to Aβ. To set up an in vitro model of the blood–brain barrier (BBB), cerebral microvascular endothelial cells were seeded on transwell inserts. After 6 days of culture, different concentrations (from 0.1 to 10 μM) of Aβ1-42 (**A**) or Aβ1-40 (**B**) oligomers were incubated in the apical compartment of the transwell system for 24 h. Then, proteins were extracted and analyzed by SDS-PAGE/WB, followed by immunoblotting of ERM proteins and its phosphorylated form (pERM). The intensity of chemiluminescent bands was estimated by Amersham 600 and normalized to β-actin. Representative blots are shown. Data are expressed as mean ± SD from three independent experiments. Statistical analysis was performed using one-way ANOVA: ** *p* < 0.01; *** *p* < 0.001.

Therefore, the phosphorylation of ERM complex (pERM) was assessed to confirm endothelial microvilli formation under Aβ exposure. After 24 h of incubation, hCMEC/D3 cells were collected and the cell lysates were analyzed by WB to detect ERM proteins. The results showed that the apical exposure of hCMEC/D3 cells to Aβ induced a significantly increase of pERM/ERM ratio of 1.4-fold for Aβ1-42 (Figure 6A) and of 1.6-fold for Aβ1-40 (Figure 6B), compared to untreated cells. Notably, the exposure to the highest Aβ concentrations tested (10 μM) did not increase the pERM/ERM ratio, possibly because of the impairment of the intracellular pathways involved in the organization of membrane protrusions. In this context, several evidence indicate that F-actin can undergo post-translational modifications (PTMs) under oxidative stimuli, leading to changes in protein polymerization [58,59,60]. While cell exposure to low levels of oxidative stress induces reversible actin modifications, high ROS concentrations promote irreversible PTM, which induce F-actin depolymerization and destabilize cell cytoskeleton, ultimately impairing cell survival [58,61]. In addition, Aβ exposure was found to increase actin aggregation and intracellular gap formation in a dose-dependent manner in endothelial cell cultures [62]. Together, these data support our findings that high Aβ concentrations may cause cytoskeleton remodeling that have repercussions on endothelial microvilli structure.

To understand if the observed cytoskeleton reorganization is directly attributable to Aβ or to Aβ-induced oxidative stress, a generic pro-oxidant agent (i.e., H_2_O_2_) was tested. The addition of a sublethal dose of H_2_O_2_ to culture medium showed a strong increase of pERM levels (+130%) compared to untreated cells, thus suggesting that a change in the pattern of microvilli protrusions correlates with the oxidative stress in endothelial cells (Supplementary Data, Figure S4A).

We also investigated if cell exposure to luminal or abluminal pro-oxidant stimuli differently affects the morphogenesis of microvilli, hCMEC/D3 cells were grown on transwell inserts and different concentrations of Aβ oligomers were added to the basolateral side of the transwell system (representing the brain side). The results showed that abluminal exposure to Aβ did not induce any significant change of pERM/ERM levels (Supplementary Data, Figure S4B), suggesting that only blood-derived stimuli can induce changes in microvilli structural organization.

**Figure 7:**
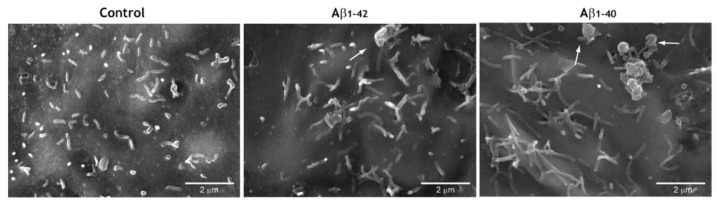
Binding of microvilli to Aβ aggregates. SEM-SE images of Aβ interaction with membrane protrusions formed by hCMEC/D3 cells when incubated with 1 μM of Aβ1-42 or Aβ1-40 oligomers for 24 h. The images show endothelial microvilli often contacting Aβ aggregates (arrows).

Curiously, the presence of Aβ oligomers was sometimes observed in connection with membrane protrusions by SEM imaging (Figure 7). It will be interesting to deepen the interaction between Aβ and endothelial microvilli in order to understand how and if this association is involved in Aβ-mediated cell toxicity. This issue deserves further investigations.

### 3.6. CNP Tropism for Endothelial Microvilli

**Figure 8:**
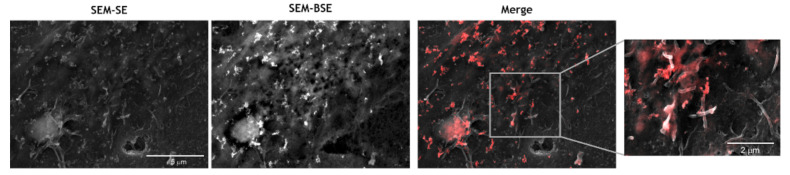
Binding of cerium oxide nanoparticles (CNP) to endothelial microvilli. SEM backscattered electrons images of CNP interaction with membrane protrusions formed by hCMEC/D3 cells after incubation with 1 μM of Aβ1-42 oligomers for 24 h. SEM secondary electrons (SEM-SE) image shows endothelial microvilli. SEM backscattered electrons (SEM-BSE) image shows CNP. The overlay of secondary electrons and backscattered electrons images (merge) and its magnified view (30kx) show the CNP (red) interaction with microvilli.

Finally, to demonstrate that CNP uptake is mediated by microvilli, hCMEC/D3 cells were exposed to 1 μM of Aβ1-42 oligomers for 24 h, followed by short-term incubation with CNP. Cells were then observed by SEM. Images showed that CNP co-localize with endothelial microvilli formed as a consequence of the stressor (i.e., Aβ) (Figure 8). This result proves that microvilli are involved in the CNP uptake, but the identification of the precise mechanism and the influence of CNP architecture need further investigations. Notably, data obtained in HeLa cells indicate that CNP interact specifically with the microvilli membrane and are subsequently internalized in endolysosomes (unpublished results), suggesting that microvilli are a target for CNP. However, there are data in literature suggesting that nanoscale particles can bind microvilli and, in turn, interact directly or indirectly with the actin filaments within microvilli can be internalized [63].

Taken together, our results suggest that Aβ induces oxidative stress on BBB endothelial cells that in turn promotes changes in the architecture of endothelial microvilli. This pathway supports particles recruitment, enhancing the uptake of CNP, which can exert their antioxidant effect.

## 4. Conclusions

Oxidative stress induces reorganization of the microvilli pattern on polarized brain capillary endothelial cells. This event fosters the internalization of antioxidant NP, which efficiently reduce ROS/RNS levels in cerebral microvascular endothelial cells.

Here we demonstrated that Aβ-induced oxidative stress promotes the formation of longer microvilli on endothelial cells, which favor the interaction of CNP with the cell surface and their internalization. The length of microvilli changed with the type of Aβ peptide (Aβ1-40 > Aβ1-42) and correlated with their cytotoxicity (Aβ1-40 > Aβ1-42). Internalized CNP efficiently reduces ROS/RNS levels in cerebral microvascular endothelial cells. It is important to highlight that the effect of CNP need to be evaluated in vivo because different issues could surface such as non-specific adhesion of CNP, long term toxicity, elimination, etc.

Therefore, NP with tropism for endothelial microvilli could be exploited to deliver exogenous antioxidants in a way proportional to the level of oxidative stress, thus reducing the local production of ROS and restoring the functionality of brain endothelium.

## Data Availability

All data is presented within the article.

## Supplementary Materials

The following are available online at https://www.mdpi.com/2076-3921/10/2/266/s1, Figure S1: A shift of 10 nm in the maximum of the emission spectra between DiI and CNP was an indication of successful intercalation of the fluorophore within the PAA hydrophobic microdomains, Figure S2: Characterisation of Aβ oligomers. (A) Aβ samples were analysed by SDS-PAGE electrophoresis and immunoblotted with anti-Aβ 6E10 antibody, followed by ECL detection. Oligomeric Aβ1-40 (lane 1), oligomeric Aβ1-42 (lane 2), monomeric Aβ1-40 (lane 3) and monomeric Aβ1-42 (lane 4) are shown. M = molecular weight size marker. (B) AFM analysis of an oligomer-enriched sample of Aβ1-40. (C) AFM analysis of an oligomer-enriched preparation of Aβ1-42, Figure S3: Cell uptake and subcellular localization of cerium oxide nanoparticles (CNP). (A) SEM backscattered electrons images of hCMEC/D3 cells incubated with CNP in the presence or absence of Aβ1-42 oligomers. Due to the compositional contrast, CNP are visible as bright spots on a darker background. The images show the presence of internalized CNP with perinuclear distribution. (B) Example of accumulation of CNP in endolysosomes, visible as round structures, Figure S4: Expression levels of phosphorylated ERM (pERM) complex by hCMEC/D3 cells after exposure to pro-oxidant agents. Cells were seeded on transwell inserts and grown for 6 days. Then, 0.5 mM H_2_O_2_ was added to the apical compartment of the transwell system (A) or increasing concentrations (from 0.1 to 10 μM) of Aβ1-42 oligomers were incubated in the basolateral compartment (mimicking the ‘brain’-side) (B) and incubated for 24 h. At the end of the treatments, proteins were extracted and subjected to western blot analysis and then to immunoblotting of ERM and its phosphorylated form. The intensity of chemiluminescent band was estimated by Amersham 600 and normalized to β-actin. Results are expressed as mean ± SD from three independent experiments. Statistical analysis was performed using one-way Anova: *** *p* < 0.001.

## References

[1] Alzheimer’s Disease International. www.alzint.org/.

[2] Hardy J.A., Higgins G.A. (1992). Alzheimer’s Disease: The Amyloid Cascade Hypothesis. Science.

[3] Selkoe D.J., Hardy J. (2016). The Amyloid Hypothesis of Alzheimer’s Disease At 25 Years. EMBO Mol. Med..

[4] Sweeney M.D., Kisler K., Montagne A., Toga A.W., Zlokovic B.V. (2018). The Role of Brain Vasculature in Neurodegenerative Disorders. Nat. Neurosci..

[5] Van de Haar H.J., Jansen J.F.A., van Osch M.J.P., van Buchem M.A., Muller M., Wong S.M., Hofman P.A.M., Burgmans S., Verhey F.R.J., Backes W.H. (2016). Neurovascular Unit Impairment in Early Alzheimer’s Disease Measured with Magnetic Resonance Imaging. Neurobiol. Aging.

[6] Park L., Wang G., Moore J., Girouard H., Zhou P., Anrather J., Iadecola C. (2014). The Key Role of Transient Receptor Potential Melastatin-2 Channels in Amyloid-Β-Induced Neurovascular Dysfunction. Nat. Commun..

[7] Park L., Wang G., Zhou P., Zhou J., Pitstick R., Previti M.L., Younkin L., Younkin S.G., Van Nostrand W.E., Cho S. (2011). Scavenger Receptor Cd36 is Essential for the Cerebrovascular Oxidative Stress and Neurovascular Dysfunction Induced by Amyloid-Beta. Proc. Natl. Acad. Sci. USA.

[8] Askarova S., Yang X., Sheng W., Sun G.Y., Lee J.C. (2011). Role of Aβ-Receptor for Advanced Glycation End Products Interaction in Oxidative Stress and Cytosolic Phospholipase A_2_ Activation in Astrocytes and Cerebral Endothelial Cells. Neuroscience.

[9] Di Marco L.Y., Venneri A., Farkas E., Evans P.C., Marzo A., Frangi A.F. (2015). Vascular Dysfunction in the Pathogenesis of Alzheimer’s Disease—A Review of Endothelium-Mediated Mechanisms and Ensuing Vicious Circles. Neurobiol. Dis..

[10] Alliev G., Priyadarshini M., Reddy V.P., Grieg N.H., Kaminsky Y., Cacabelos R., Ashraf G.M., Jabir N.R., Kamal M.A., Nikolenko V.N. (2014). Oxidative Stress Mediated Mitochondrial and Vascular Lesions as Markers in the Pathogenesis of Alzheimer Disease. Curr. Med. Chem..

[11] Enciu A.M., Gherghiceanu M., Popescu O.B. (2013). Triggers and Effectors of Oxidative Stress at Blood-Brain Barrier Level: Relevance for Brain Ageing and Neurodegeneration. Oxid. Med. Cell. Longev..

[12] Bulbarelli A., Lonati E., Brambilla A., Orlando A., Cazzaniga E., Piazza F., Ferrarese C., Masserini M., Sancini G. (2012). Aβ42 Production in Brain Capillary Endothelial Cells after Oxygen and Glucose Deprivation. Mol. Cell. Neurosci..

[13] Kalaria R., Premkumar D., Pax A., Cohen D., Lieberburg I. (1996). Production and Increased Detection of Amyloid Beta Protein and Amyloidogenic Fragments in Brain Microvessels, Meningeal Vessels and Choroid Plexus in Alzheimer’s Disease. Mol. Brain Res..

[14] Martinelli C., Pucci C., Battaglini M., Marino A., Ciofani G. (2020). Antioxidants and Nanotechnology: Promises and Limits of Potentially Disruptive Approaches in the Treatment of Central Nervous System Diseases. Adv. Healthc. Mater..

[15] Gilgun-Sherki Y., Melamed E., Offen D. (2001). Oxidative Stress Induced-Neurodegenerative Diseases: The Need for Antioxidants That Penetrate the Blood Brain Barrier. Neuropharmacology.

[16] Rakotoarisoa M., Angelov B., Garamus V.M., Angelova A. (2019). Curcumin- and Fish Oil-Loaded Spongosome and Cubosome Nanoparticles with Neuroprotective Potential against H2O2-Induced Oxidative Stress in Differentiated Human SH-SY5Y Cells. ACS Omega.

[17] Rakotoarisoa M., Angelov B., Espinoza S., Khakurel K., Bizien T., Angelova A. (2019). Cubic Liquid Crystalline Nanostructures Involving Catalase and Curcumin: BioSAXS Study and Catalase Peroxidatic Function after Cubosomal Nanoparticle Treatment of Differentiated SH-SY5Y Cells. Molecules.

[18] Santonocito D., Sarpietro M.G., Carbone C., Panico A., Campisi A., Siciliano E.A., Sposito G., Castelli F., Puglia C. (2020). Curcumin Containing PEGylated Solid Lipid Nanoparticles for Systemic Administration: A Preliminary Study. Molecules.

[19] Celardo I., Pedersen J.Z., Traversa E., Ghibelli L. (2011). Pharmacological Potential of Cerium Oxide Nanoparticles. Nanoscale.

[20] Ferraro D., Tredici I.G., Ghigna P., Castillo-Michel H., Falqui A., Di Benedetto C., Alberti G., Ricci V., Anselmi-Tamburini U., Sommi P. (2017). Dependence of the Ce(iii)/Ce(iv) Ratio on Intracellular Localization in Ceria Nanoparticles Internalized by Human Cells. Nanoscale.

[21] D’Angelo B., Santucci S., Benedetti E., Di Loreto S., Phani R.A., Falone S., Amicarelli F., Ceru M.P., Cimini A. (2009). Cerium Oxide Nanoparticles Trigger Neuronal Survival in A Human Alzheimer Disease Model by Modulating Bdnf Pathway. Curr. Nanosci..

[22] Kwon H.J., Moon-Yong C., Dokyoon K., Dong K.K., Min S., Kwangsoo S., Taeghwan H., Inhee M.J. (2016). Mitochondria-Targeting Ceria Nanoparticles as Antioxidants for Alzheimer’s Disease. ACS Nano.

[23] Dowding J.M., Song W., Bossy K., Karakoti A., Kumar A., Kim A., Bossy B., Seal S., Ellisman M.H., Perkins G. (2014). Cerium Oxide Nanoparticles Protect Against Aβ-Induced Mitochondrial Fragmentation and Neuronal Cell Death. Cell Death Differ..

[24] Li M., Peng S., Can X., Jinsong R., Xiaogang Q. (2013). Cerium Oxide Caged Metal Chelator: Anti-Aggregation and Anti-Oxidation Integrated H2o2-Responsive Controlled Drug Release for Potential Alzheimer’s Disease Treatment. Chem. Sci..

[25] Asati A., Santra S., Kaittanis C., Perez J.M. (2010). Surface-Charge-Dependent Cell Localization and Cytotoxicity of Cerium Oxide Nanoparticles. ACS Nano.

[26] Santra S., Kaittanis C., Grimm J., Perez J.M. (2009). Drug/Dye-Loaded, Multifunctional Iron Oxide Nanoparticles for Combined Targeted Cancer Therapy and Dual Optical/Magnetic Resonance Imaging. Small.

[27] Mancini S., Minniti S., Gregori M., Sancini G., Cagnotto A., Couraud P.O., Ordóñez-Gutiérrez L., Wandosell F., Salmona M., Re F. (2016). The Hunt for Brain Aβ Oligomers by Peripherally Circulating Multi-Functional Nanoparticles: Potential Therapeutic Approach for Alzheimer Disease. Nanomed. Nanotechnol. Biol. Med..

[28] Dal Magro R., Simonelli S., Cox A., Formicola B., Corti R., Cassina V., Nardo L., Mantegazza F., Salerno D., Grasso G. (2019). The Extent of Human Apolipoprotein A-I Lipidation Strongly Affects the β-Amyloid Efflux Across the Blood-Brain Barrier in Vitro. Front. Neurosci..

[29] Nardo L., Re F., Brioschi S., Cazzaniga E., Orlando A., Minniti S., Lamperti M., Gregori M., Cassina V., Brogioli D. (2016). Fluorimetric Detection of the Earliest Events in Amyloid β Oligomerization and Its Inhibition by Pharmacologically Active Liposomes. Biochim. Biophys. Acta (BBA) Gen. Subj..

[30] Weksler B.M., Romero I.A., Couraud P.O. (2013). The hCMEC/D3 Cell Line as A Model of the Human Blood Brain Barrier. Fluids Barriers CNS.

[31] Gregori M., Orlando A., Re F., Sesana S., Nardo L., Salerno D., Mantegazza F., Salvati E., Zito A., Malavasi F. (2016). Novel Antitransferrin Receptor Antibodies Improve the Blood-Brain Barrier Crossing Efficacy of Immunoliposomes. J. Pharm. Sci..

[32] Kuo Y.M., Kokjohn T.A., Kalback W., Luehrs D., Galasko D.R., Chevallier N., Koo E.H., Emmerling M.R., Roher A.E. (2000). Amyloid-Beta Peptides Interact with Plasma Proteins and Erythrocytes: Implications for Their Quantitation in Plasma. Biochem. Biophys. Res. Commun..

[33] Cox A., Andreozzi P., Dal Magro R., Fiordaliso F., Corbelli A., Talamini L., Chinello C., Raimondo F., Magni F., Tringali M. (2018). Evolution of Nanoparticle Protein Corona across the Blood-Brain Barrier. ACS Nano.

[34] Formicola B., Dal Magro R., Montefusco-Pereira C.V., Lehr C.M., Koch M., Russo L., Grasso G., Deriu M.A., Danani A., Bourdoulous S. (2019). The Synergistic Effect of Chlorotoxin-mApoE in Boosting Drug-Loaded Liposomes Across The BBB. J. Nanobiotechnol..

[35] Pezzini I., Marino A., Del Turco S., Nesti C., Doccini S., Cappello V., Gemmi M., Parlanti P., Santorelli F.M., Mattoli V. (2017). Cerium Oxide Nanoparticles: The Regenerative Redox Machine in Bioenergetic Imbalance. Nanomedicine.

[36] Hanafy B.I., Cave G.W.V., Barnett Y., Pierscionek B. (2020). Treatment of Human Lens Epithelium with High Levels of Nanoceria Leads to Reactive Oxygen Species Mediated Apoptosis. Molecules.

[37] Tomic J.L., Pensalfini A., Head E., Glabe C.G. (2009). Soluble Fibrillar Oligomer Levels Are Elevated in Alzheimer’s Disease Brain and Correlate with Cognitive Dysfunction. Neurobiol. Dis..

[38] Lee S.J., Nam E., Lee H.J., Savelieff M.G., Lim M.H. (2017). Towards an Understanding of Amyloid-Β Oligomers: Characterization, Toxicity Mechanisms, and Inhibitors. Chem. Soc. Rev..

[39] Xu J., Chen S., Ku G., Ahmed S.H., Xu J., Chen H., Hsu C.Y. (2001). Amyloid Beta Peptide-Induced Cerebral Endothelial Cell Death Involves Mitochondrial Dysfunction and Caspase Activation. J. Cereb. Blood Flow Metab..

[40] Thal D.R., Griffin W.S., de Vos R.A., Ghebremedhin E. (2008). Cerebral Amyloid Angiopathy and Its Relationship to Alzheimer’s Disease. Acta Neuropathol..

[41] Kirkitadze M.D., Bitan G., Teplow D.B. (2002). Paradigm Shifts in Alzheimer’s Disease and Other Neurodegenerative Disorders: The Emerging Role of Oligomeric Assemblies. J. Neurosci. Res..

[42] Durán-Prado M., Frontiñán J., Santiago-Mora R., Peinado J.R., Parrado-Fernández C., Gómez-Almagro M.V., Moreno M., López-Domínguez J.A., Villalba J.M., Alcaín F.J. (2014). Coenzyme Q10 Protects Human Endothelial Cells from Β-Amyloid Uptake and Oxidative Stress-Induced Injury. PLoS ONE.

[43] Tscheka C., Hittinger M., Lehr C.M., Schneider-Daum N., Schneider M. (2015). Macrophage Uptake of Cylindrical Microparticles Investigated with Correlative Microscopy. Eur. J. Pharm. Biopharm..

[44] Dal Magro R., Ornaghi F., Cambianica I., Beretta S., Re F., Musicanti C., Rigolio R., Donzelli E., Canta A., Ballarini E. (2017). ApoE-Modified Solid Lipid Nanoparticles: A Feasible Strategy to Cross the Blood-Brain Barrier. J. Control. Release.

[45] Cox A., Vinciguerra D., Re F., Magro R.D., Mura S., Masserini M., Couvreur P., Nicolas J. (2019). Protein-Functionalized Nanoparticles Derived from End-Functional Polymers and Polymer Prodrugs for Crossing the Blood-Brain Barrier. Eur. J. Pharm. Biopharm..

[46] Bana L., Minniti S., Salvati E., Sesana S., Zambelli V., Cagnotto A., Orlando A., Cazzaniga E., Zwart R., Scheper W. (2014). Liposomes Bi-Functionalized with Phosphatidic Acid and an Apoe-Derived Peptide Affect Aβ Aggregation Features and Cross The Blood-Brain-Barrier: Implications for Therapy of Alzheimer Disease. Nanomedicine.

[47] Songjiang Z., Lixiang W. (2009). Amyloid-Beta Associated with Chitosan Nano-Carrier Has Favorable Immunogenicity and Permeates The Bbb. Aaps Pharmscitech.

[48] Gabbiani G., Majno G. (1969). Endothelial Microvilli in the Vessels of The Rat Gasserian Ganglion and Testis. Z. Zellforsch. Mikrosk. Anat..

[49] Makarov V., Zueva L., Sanabria P., Wessinger W.D., Golubeva T., Khmelinskii I., Inyushin M. (2015). On the Role of the Blood Vessel Endothelial Microvilli in the Blood Flow in Small Capillaries. J. Biophys..

[50] Carman C.V., Jun C.D., Salas A., Springer T.A. (2003). Endothelial Cells Proactively form Microvilli-Like Membrane Projections Upon Intercellular Adhesion Molecule 1 Engagement of Leukocyte LFA-1. J. Immunol..

[51] Arita-Okubo S., Kim-Kaneyama J.R., Lei X.F., Fu W.G., Ohnishi K., Takeya M., Miyauchi A., Honda H., Itabe H., Miyazaki T. (2015). Role of Hic-5 in the Formation of Microvilli-Like Structures and the Monocyte-Endothelial Interaction that Accelerates Atherosclerosis. Cardiovasc. Res..

[52] Eugène E., Hoffmann I., Pujol C., Couraud P.O., Bourdoulous S., Nassif X. (2002). Microvilli-Like Structures Are Associated with the Internalization of Virulent Capsulated Neisseria Meningitidis into Vascular Endothelial Cells. J. Cell Sci..

[53] Dietrich W.D., Busto R., Ginsberg M.D. (1984). Cerebral Endothelial Microvilli: Formation Following Global Forebrain Ischemia. J. Neuropathol. Exp. Neurol..

[54] Lossinsky A.S., Vorbrodt A.W., Wisniewski H.M. (1995). Scanning and Transmission Electron Microscopic Studies of Microvascular Pathology in the Osmotically Impaired Blood-Brain Barrier. J. Neurocytol..

[55] Gautreau A., Louvard D., Arpin M. (2000). Morphogenic Effects of Ezrin Require a Phosphorylation-Induced Transition from Oligomers to Monomers at the Plasma Membrane. J. Cell Biol..

[56] Yamane J., Ohnishi H., Sasaki H., Narimatsu H., Ohgushi H., Tachibana K. (2011). Formation of Microvilli and Phosphorylation of Erm Family Proteins by Cd43, A Potent Inhibitor for Cell Adhesion: Cell Detachment Is a Potential Cue for Erm Phosphorylation and Organization of Cell Morphology. Cell Adhes. Migr..

[57] Yonemura S., Hirao M., Doi Y., Takahashi N., Kondo T., Tsukita S. (1998). Ezrin/Radixin/Moesin (Erm) Proteins Bind to a Positively Charged Amino Acid Cluster in The Juxta-Membrane Cytoplasmic Domain of CD44, CD43, and ICAM-2. J. Cell Biol..

[58] Varland S., Vandekerckhove J., Drazic A. (2019). Actin Post-Translational Modifications: The Cinderella of Cytoskeletal Control. Trends Biochem. Sci..

[59] Hung R.J., Pak C.W., Terman J.R. (2011). Direct Redox Regulation of F-Actin Assembly and Disassembly by Mical. Science.

[60] Kommaddi R.P., Tomar D.S., Karunakaran S., Bapat D., Nanguneri S., Ray A., Schneider B.L., Nair D., Ravindranath V. (2019). Glutaredoxin1 Diminishes Amyloid Beta-Mediated Oxidation of F-Actin and Reverses Cognitive Deficits in an Alzheimer’s Disease Mouse Model. Antioxid. Redox Signal..

[61] Wong S.W., Sun S., Cho M., Lee K.K., Mak A.F. (2015). H_2_O_2_ Exposure Affects Myotube Stiffness and Actin Filament Polymerization. Ann. Biomed. Eng..

[62] Nagababu E., Usatyuk P.V., Enika D., Natarajan V., Rifkind J.M. (2009). Vascular Endothelial Barrier Dysfunction Mediated by Amyloid-Beta Proteins. J. Alzheimers Dis..

[63] Orr G., Panther D.J., Phillips J.L., Tarasevich B.J., Dohnalkova A., Hu D., Teeguarden J.G., Pounds J.G. (2007). Submicrometer and Nanoscale Inorganic Particles Exploit the Actin Machinery to Be Propelled along Microvilli-Like Structures into Alveolar Cells. ACS Nano.

