# Supplementary material for: Oxidative Stress Boosts the Uptake of Cerium Oxide Nanoparticles by Changing Brain Endothelium Microvilli Pattern
Source: Antioxidants (Basel). 2021 Feb 9;10(2):266. doi: 10.3390/antiox10020266 (PMC7916071; doi:10.3390/antiox10020266)
Supplement: Supplementary file 1 [file antioxidants-10-00266-s001.pdf]

Supplementary data

Oxidative stress boosts the uptake of cerium oxide nanoparticles by changing brain endothelium microvilli pattern

*Roberta Dal Magro [a] *, Agostina Vitali [b], Stefano Fagioli [a], Alberto Casu [c], Andrea Falqui [c], Beatrice Formicola [a], Lorenzo Taiarol [a], Valeria Cassina [a] , Claudia Adriana Marrano [a], Francesco Mantegazza [a], Umberto Anselmi-Tamburini [b], Patrizia Sommi [d ‡], Francesca Re [a ‡]*

[a] School of Medicine and Surgery, Nanomedicine Center NANOMIB, University of Milano-Bicocca, 20900 Monza, Italy

[b] Department of Chemistry, University of Pavia, 27100 Pavia, Italy

[c] Biological and Environmental Sciences and Engineering Division, NABLA Lab, King Abdullah University of Science and Technology (KAUST), 23955-6900 Thuwal, Saudi Arabia

[d] Department of Molecular Medicine, Human Physiology Unit, University of Pavia, 27100 Pavia, Italy.

‡ Equally contributed authors

* **Corresponding author**: Roberta Dal Magro - School of Medicine and Surgery, Nanomedicine Center NANOMIB, University of Milano-Bicocca, 20900 Monza, Italy.
Email: roberta.dalmagro@unimib.it

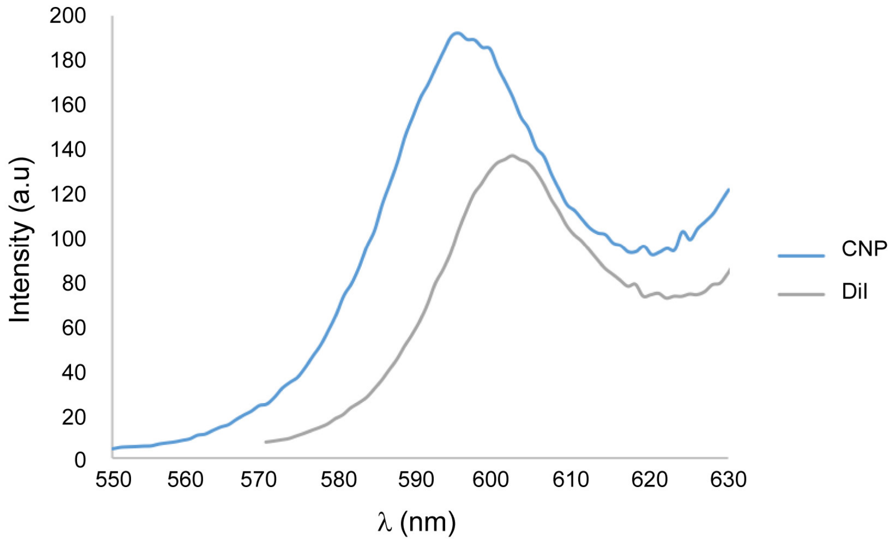

Figure S1. A shift of 10 nm in the maximum of the emission spectra between DiI and CNP was an indication of successful intercalation of the fluorophore within the PAA hydrophobic microdomains.

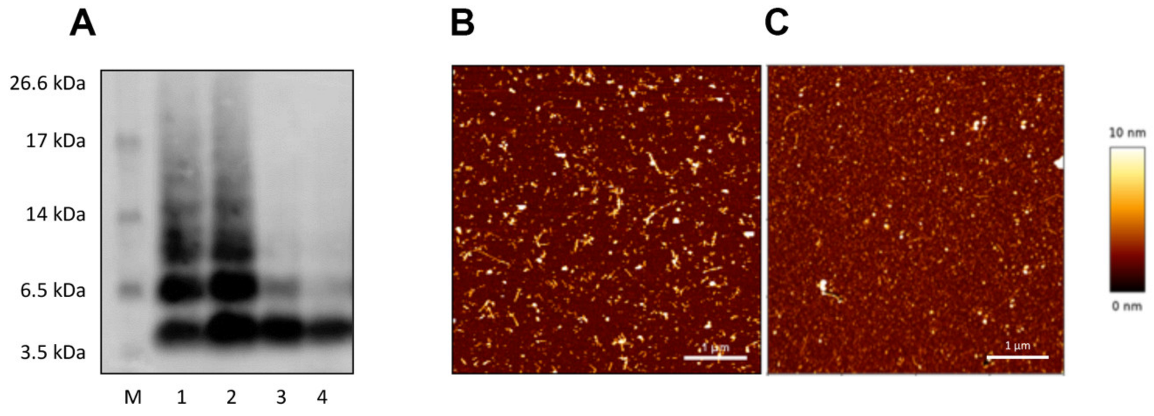

Figure S2. Characterisation of Aβ oligomers. **(A)** Aβ samples were analysed by SDS-PAGE electrophoresis and immunoblotted with anti-Aβ 6E10 antibody, followed by ECL detection. Oligomeric $Aβ_{1-40}$ (lane 1), oligomeric $Aβ_{1-42}$ (lane 2), monomeric $Aβ_{1-40}$ (lane 3) and monomeric $Aβ_{1-42}$ (lane 4) are shown. M = molecular weight size marker. **(B)** AFM analysis of an oligomer-enriched sample of $Aβ_{1-40}$. **(C)** AFM analysis of an oligomer-enriched preparation of $Aβ_{1-42}$.

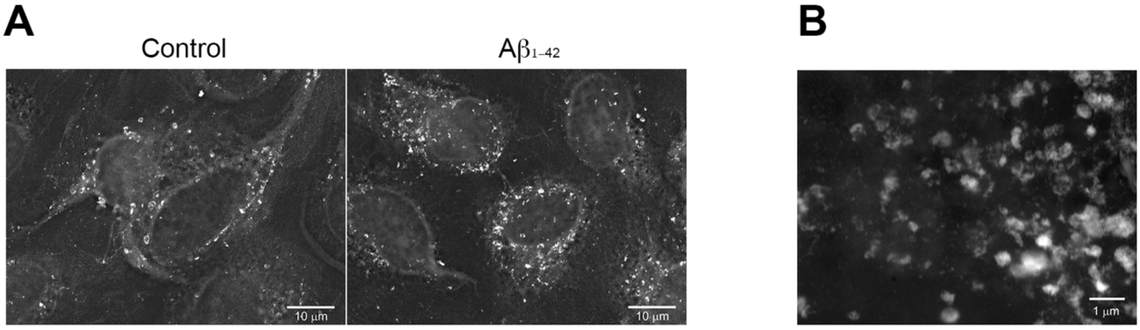

Figure S3. Cell uptake and subcellular localization of cerium oxide nanoparticles (CNP). **(A)** SEM backscattered electrons images of hCMEC/D3 cells incubated with CNP in the presence or absence of Aβ_{1-42} oligomers. Due to the compositional contrast, CNP are visible as bright spots on a darker background. The images show the presence of internalized CNP with perinuclear distribution. **(B)** Example of accumulation of CNP in endolysosomes, visible as round structures.

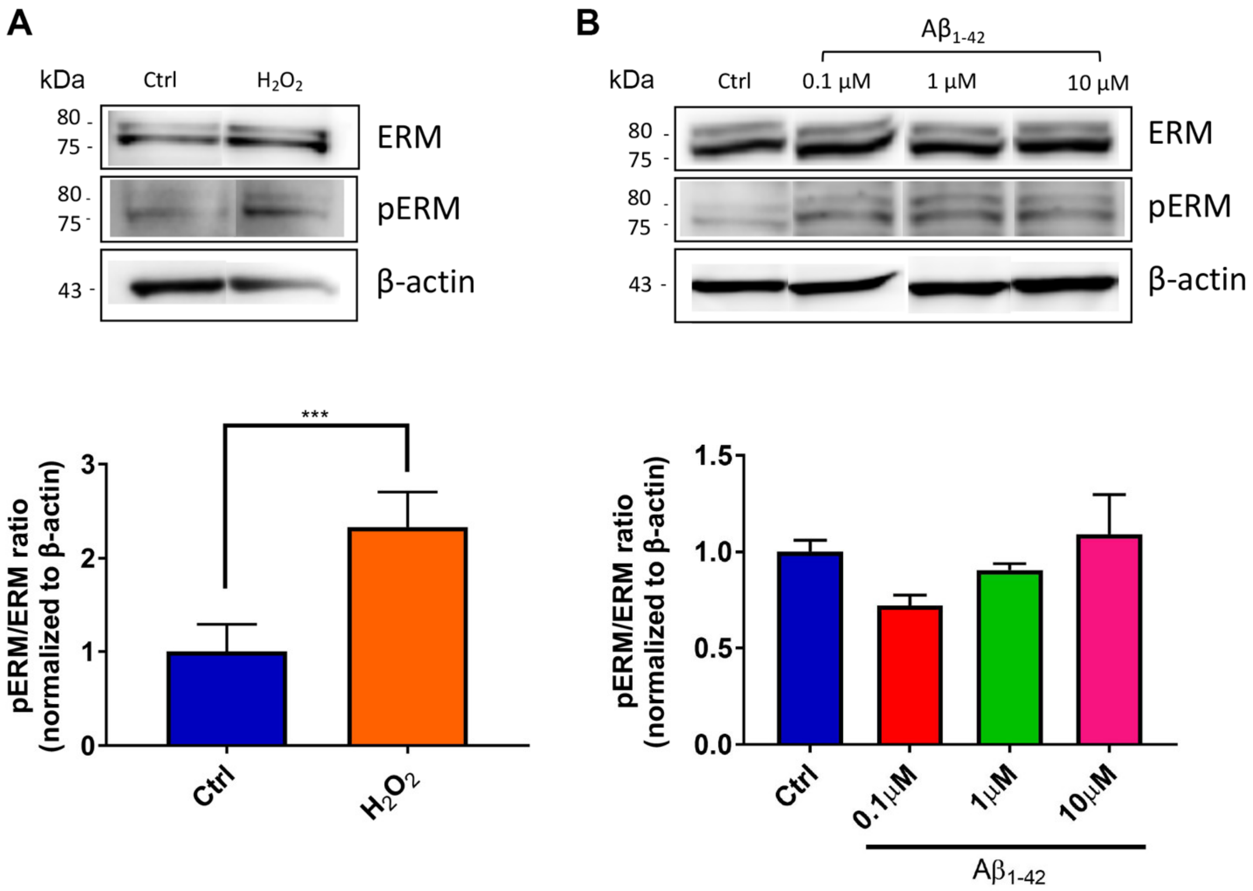

Figure S4. Expression levels of phosphorylated ERM (pERM) complex by hCMEC/D3 cells after exposure to pro-oxidant agents. Cells were seeded on transwell inserts and grown for 6 days. Then, 0.5 mM H_2O_2 was added to the apical compartment of the transwell system **(A)** or increasing concentrations (from 0.1 to 10 μM) of $A\beta_{1\text{-}42}$ oligomers were incubated in the basolateral compartment (mimicking the 'brain'-side) **(B)** and incubated for 24 h. At the end of the treatments, proteins were extracted and subjected to western blot analysis and then to immunoblotting of ERM and its phosphorylated form. The intensity of chemiluminescent band was estimated by Amersham 600 and normalized to β-actin. Results are expressed as mean ± SD from three independent experiments. Statistical analysis was performed using one-way Anova: ***$p<0.001$.
